# Two Cases of Cutaneous Nocardiosis After a Natural Disaster

**DOI:** 10.7759/cureus.6278

**Published:** 2019-12-02

**Authors:** Sarah Al-Obaydi, James DeMaio

**Affiliations:** 1 Internal Medicine, Blake Medical Center, Bradenton, USA

**Keywords:** nocardia brasiliensis, natural disasters, lymphadenitis, hurricane, skin and soft tissue infections, cutaneous nocardiosis

## Abstract

Skin and soft tissue infections have been well-documented after natural disasters; however, to the best of our knowledge, Nocardia brasiliensis (N. brasiliensis) is not included in the Centers for Disease Control and Prevention (CDC) list of environmental pathogens associated with tropical storms. In this report, we describe two cases of N. brasiliensis lymphadenitis that occurred four to six weeks after Hurricane Irma hit Manatee County, Florida. Since N. brasiliensis skin and soft tissue infections are typically very uncommon in our patient population, we concluded that cases of N. brasiliensis could increase after tropical storms, and we suggest that this pathogen is to be included in the CDC’s list of environmental pathogens associated with natural disasters.

## Introduction

An increase in skin and soft tissue infections has been well-documented after natural disasters [1-2]. The causative organisms may be either part of the patient’s skin flora or environmental pathogens that have contaminated wounds. Previously described environmental pathogens include Vibrio vulnificus, Aeromonas species, non-tuberculous mycobacteria, and mucormycosis. However, a review of the peer-reviewed literature revealed no previously reported cases of Nocardia brasiliensis infection associated with natural disasters. This is surprising since N. brasiliensis lymphadenitis classically occurs after trauma involving either plant material or soil. We present two cases of N. brasiliensis lymphadenitis that occurred in West Florida after being hit by Hurricane Irma in September 2017.

## Case presentation

Case 1

A 75-year-old immune-competent male with a medical history significant for essential hypertension, gastroesophageal reflux disease, and hyperlipidemia presented to the emergency department approximately four weeks after Hurricane Irma with an abscess on his left forearm and pain and erythema extending from his forearm to his axilla. He did not have any associated fever or chills. The patient thought that the abscess might have started after he injured his arm while retrieving a golf ball 10 days before the presentation. He was admitted to the hospital for presumed cellulitis and lymphadenitis.

**Figure 1:**
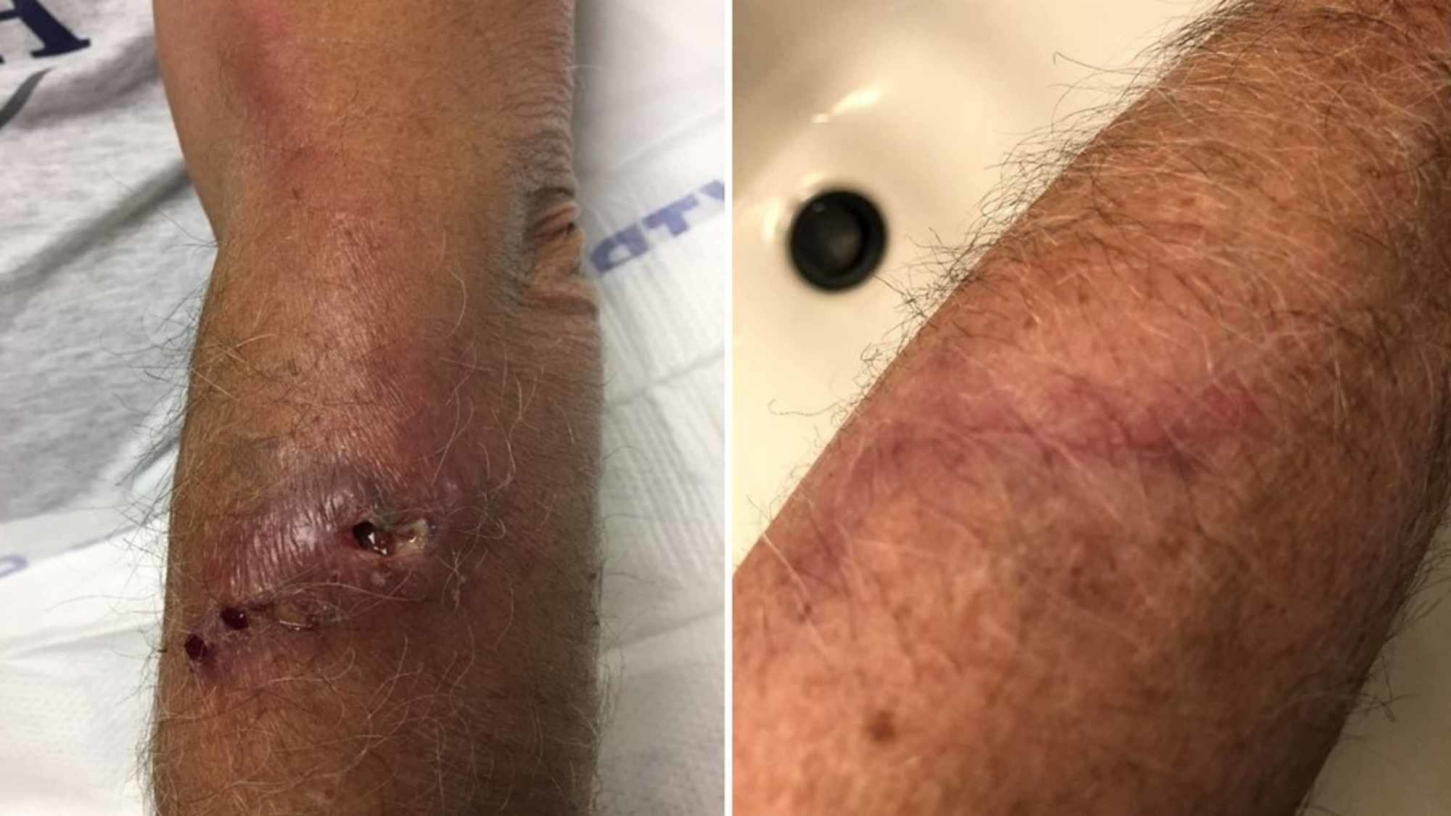
Nocardia lesions before and after treatment

Initial laboratory testing, including a complete blood count and comprehensive metabolic panel, was non-contributory. The patient was started empirically on intravenous vancomycin. However, despite antibiotic treatment, the lymph nodes began to suppurate, and the patient was taken to the operating room for incision and debridement. Intraoperative gram stain revealed gram-positive rods and cultures grew N. brasiliensis. The patient was switched to oral sulfamethoxazole/trimethoprim and was discharged home to continue on the antibiotic regimen for six weeks with close follow-up at the infectious disease clinic until there was a resolution of the lesions (Figure 1). The patient provided written consent to publish the images associated with his case description.

Case 2

A 70-year-old immune-competent male with a past medical history of ischemic coronary artery disease, hypertension, diabetes mellitus type II, and hypothyroidism presented to the emergency department about seven weeks post-Hurricane Irma after injuring his finger while trimming a palm branch five days before presentation. The patient reported low-grade fevers, mild chills, and swelling in his left arm with nodules tracking up to his axilla. Physical examination showed a draining abscess on the dorsal surface of his left ring finger and cellulitis and lymphadenitis extending up to his entire arm. No necrotic lesions were noted, but the nodules along the arm were very tender to touch with extensive surrounding cellulitis.

Laboratory testing, including a complete blood count and comprehensive metabolic panel, was non-revealing, except for mild leukocytosis with a left shift. Since this patient presented within a couple of weeks after Hurricane Irma, and given that we encountered a similar presentation earlier (Case 1), our suspicion that this might be another case of Nocardia was high and the decision was made to take the patient to the OR immediately for incision and drainage. He was started on intravenous vancomycin and ceftriaxone to cover both methicillin-resistant Staphylococcus aureus (MRSA) and Nocardia. Intraoperative cultures grew N. brasiliensis. The patient was switched to oral sulfamethoxazole/trimethoprim alone to continue for six weeks after discharge, which resulted in the resolution of the lesion.

## Discussion

Our two patients were admitted with nearly identical clinical presentations within weeks of each other shortly after Hurricane Irma. Hurricane Irma hit West Florida in September 2017 and generated sustained winds exceeding 80 mph in West Florida, leading to extensive soil and plant disturbance in the area. The subsequent surface contamination with soil particles likely placed our patients at an increased risk of infection.

We performed a search of the electronic medical records (EMRs) of patients admitted at our facility, as well as of those seen at our infectious disease outpatient clinic between the years of 2006 and 2017 prior to Hurricane Irma, and we were only able to identify eight cases of N. brasiliensis skin and soft tissue infections. Upon further review of the EMRs of patients across the 17 other affiliated hospitals in West Florida between 2015 and 2019, and we identified two additional microbiologically-confirmed cases. Our review highlighted N. brasiliensis as an uncommon diagnosis in our facility (< 1 case per year) and strengthens the relationship between Hurricane Irma and the occurrence of the two cases presented. A systematic review of the PubMed database identified no such cases.

Prior reviews have listed numerous pathogens associated with skin and soft tissue infection after natural disasters. According to the Centers for Disease Control and Prevention (CDC), MRSA and Vibrio vulnificus [1] are the most common culprits. A review published by Cann et al. [2] found that 28.4% of the cases reported after extreme water-related weather events were due to V. vulnificus. 

After Hurricane Katrina in 2005, the CDC published a report of 18 wound infections associated with Vibrio in the affected states; of these, 82% of the infections were due to V. vulnificus [3]. Infections with organisms that inhabit soil, such as fungi, have also been reported after natural disasters as well. For instance, a nine-fold increase in the incidence of coccidioidomycosis infection was noted after the 1994 earthquake in Southern California. This was thought to be due to the dispersion of spores of Coccidioides by the massive dust clouds [4].

To our knowledge, no published review examined the occurrence of N. brasiliensis skin and soft tissue infections after natural disasters, nor are there posted recommendations including this organism in the list of environmental pathogens associated with tropical storms.

We conjecture that the sustained wind generated by Hurricane Irma led to extensive soil and plant disturbance in the area, which resulted in surface contamination with the pathogen.

## Conclusions

Based on the experience we had from both patients, we suggest that Nocardia brasiliensis be included in the list of environmental pathogens associated with tropical storms for clinicians to be aware of its importance after natural disasters. We also believe that future research is warranted to test our hypothesis and to identify more pathogens that could potentially be associated with tropical storms.

## References

[1] CDC. (Jan 2019 (2019). Infectious Disease After a Disaster. http://www.cdc.gov/disasters/disease/infectious.html.

[2] Cann KF, Thomas DR, Salmon RL, Wyn-Jones AP, Kay D (2013). Extreme water-related weather events and waterborne disease. Epidemiol Infect.

[3] CDC. (Sept 2005 (2019). Vibrio Illnesses After Hurricane Katrina --- Multiple States, August--September 2005. http://www.cdc.gov/mmwr/preview/mmwrhtml/mm54d914a1.htm.

[4] Watson JT, Gayer M, Connolly MA (2007). Epidemics after natural disasters. Emerg Infect Dis.

